# Arts and creativity interventions for improving health and wellbeing in older adults: a systematic literature review of economic evaluation studies

**DOI:** 10.1186/s12889-023-17369-x

**Published:** 2023-12-13

**Authors:** Grainne Crealey, Laura McQuade, Roger O’Sullivan, Ciaran O’Neill

**Affiliations:** 1 Clinical Costing Solutions, Belfast, BT15 4EB UK; 2 Institute of Public Health, 200 South Circular Road, Dublin 8, D08 NH90 Ireland; 3 Centre for Public Health, Institute of Clinical Sciences, Royal Victoria Hospital, Belfast, BT12 6BA UK

**Keywords:** Arts, Creativity, Economic evaluation, Health, Wellbeing, Older adults

## Abstract

**Background:**

As the population ages, older people account for a larger proportion of the health and social care budget. A significant body of evidence suggests that arts and creativity interventions can improve the physical, mental and social wellbeing of older adults, however the value and/or cost-effectiveness of such interventions remains unclear.

**Methods:**

We systematically reviewed the economic evidence relating to such interventions, reporting our findings according to PRISMA guidelines. We searched bibliographic databases (MEDLINE, EMBASE, Econlit and Web of Science and NHSEED), trial registries and grey literature. No language or temporal restrictions were applied. Two screening rounds were conducted independently by health economists experienced in systematic literature review. Methodological quality was assessed, and key information extracted and tabulated to provide an overview of the published literature. A narrative synthesis without meta-analysis was conducted.

**Results:**

Only six studies were identified which provided evidence relating to the value or cost-effectiveness of arts and creativity interventions to improve health and wellbeing in older adults. The evidence which was identified was encouraging, with five out of the six studies reporting an acceptable probability of cost-effectiveness or positive return on investment (ranging from £1.20 to over £8 for every £1 of expenditure). However, considerable heterogeneity was observed with respect to study participants, design, and outcomes assessed. Of particular concern were potential biases inherent in social value analyses.

**Conclusions:**

Despite many studies reporting positive health and wellbeing benefits of arts and creativity interventions in this population, we found meagre evidence on their value or cost-effectiveness. Such evidence is costly and time-consuming to generate, but essential if innovative non-pharmacological interventions are to be introduced to minimise the burden of illness in this population and ensure efficient use of public funds. The findings from this review suggests that capturing data on the value and/or cost-effectiveness of such interventions should be prioritised; furthermore, research effort should be directed to developing evaluative methods which move beyond the confines of current health technology assessment frameworks, to capture a broader picture of ‘value’ more applicable to arts and creativity interventions and public health interventions more generally.

**PROSPERO registration:**

CRD42021267944 (14/07/2021).

**Supplementary Information:**

The online version contains supplementary material available at 10.1186/s12889-023-17369-x.

## Background

The number and proportion of older adults in the population has increased in virtually every country in the world over past decades [1]. In 2015, there were around 901 million people aged 60 years and over worldwide, by 2030, this will have increased to 1.4 billion [2]. An ageing population is one of the greatest successes of public health but it has implications for economies in numerous ways: slower labour force growth; working-age people will have to make greater provisions in welfare payments for older people who are no longer economically active; provisions for increased long-term care; and, society must adjust to the changing needs, expectations and capabilities of an expanding group of its citizens.

The Covid-19 pandemic shone an uncompromising light on the health and social care sector, highlighting the seriousness of gaps in policies, systems and services. It also focused attention on the physical and mental health consequences of loneliness and social isolation. To foster healthy ageing and improve the lives of older people, their families and communities, sustained and equitable investment in health and wellbeing is required [3]. The prevailing model of health and social care which is based ostensibly on formal care provision is unlikely to be sustainable over the longer term. New models, which promote healthy ageing and recognise the need for increasing reliance on self-care are required, as will be evidence of their effectiveness, cost-effectiveness and scalability.

Arts and creativity interventions (ACIs) can have positive effects on health and well-being, as several reviews have shown [4, 5]. For older people, ACI’s can enhance wellbeing [6–9], quality of life [10, 11] and cognitive function [12–16]. They can also foster social cohesion [17–19] and reduce social disparities and injustices [20]; promote healthy behaviour; prevent ill health (including enhancing well-being and mental health) [21–25], reducing cognitive decline [26, 27], frailty [28–33] and premature mortality [34–38]); support people with stroke [39–42]; degenerative neurological disorders and dementias and support end of life care [43, 44]. Moreover, ACIs can benefit not only individuals, but also others, such as supporting the well-being of formal and informal carers, enriching our knowledge of health, and improving clinical skills [4, 5].

The benefits of ACIs have also been acknowledged at a governmental level by those responsible for delivering health and care services: The UK All-Party Parliamentary Special Interest group on Arts, Health and Wellbeing produced a comprehensive review of creative intervention for health and wellbeing [45]. This report contained three key messages: that the arts can keep us well, aid recovery and support longer better lived lives; they can help meet major challenges facing health and social care; and that the arts can save money for the health service and social care.

Despite robust scientific evidence and governmental support, no systematic literature review has collated the evidence with respect to the value, cost or cost-effectiveness of such interventions. Our objective was to assess the economic impact of ACIs aimed at improving the health and wellbeing of older adults; to determine the range and quality of available studies; identify gaps in the evidence-base; and guide future research, practice and policy.

## Methods

A protocol for this review was registered at PROSPERO, an international prospective register of systematic reviews (Registration ID CRD42021267944). We used pre-determined criteria for considering studies to include in the review, in terms of types of studies, participant and intervention characteristics.

The review followed the five-step approach on how to prepare a Systematic Review of Economic Evaluations (SR-EE) for informing evidence-based healthcare decisions [46–48]. Subsequent to developing and registering the protocol, the International Society for Pharmacoeconomic Outcomes and Research (ISPOR) published a good practice task force report for the critical appraisal of systematic reviews with costs and cost-effectiveness outcomes (SR-CCEOs) [49]. This was also used to inform the conduct of this review.

### Eligibility criteria

Full economic evaluations are regarded as the optimal type of evidence for inclusion in a SR-EE [46], hence cost-minimisation analyses (CMA), cost-effectiveness analyses (CEA), cost-utility analyses (CUA) and cost–benefit analyses (CBA) were included. Social value analyses were also included as they are frequently used to inform decision-making and commissioning of services within local government. Additionally, they represent an important intermediate stage in our understanding of the costs and consequences of public health interventions, where significant challenges exist with regard to performing full evaluations [50–53].

### Development of search strategies

The population (P), intervention (I), comparator (C) and outcomes (O) (PICO) tool provided a framework for development of the search strategy. Studies were included if participants were aged 50 years or older (or if the average age of the study population was 50 years or over). Interventions could relate to performance art (dance, singing, theatre, drama etc.), creative and visual arts (painting, sculpture, art making and design), or creative writing (writing narratives, poetry, storytelling). The intervention had to be active (for example, creating art as opposed to viewing art; playing an instrument as opposed to listening to music). The objective of the intervention had to be to improve health and wellbeing; it had to be delivered under the guidance of a professional; delivered in a group setting and delivered on more than one occasion. No restrictions were placed on the type of comparator(s) or the type of outcomes captured in the study. We deliberately limited the study to professionally led activities to provide a sharper distinction between social events where arts and creativity may occur and arts and creativity interventions per se. We set no language restriction nor a restriction on the date from which studies were reported.

### Search methods

PRESS (peer-review electronic search strategies) guidelines informed the design our search strategy [54, 55] and an information specialist adapted the search terms (outlined in Table S1) for the following electronic bibliographic databases: MEDLINE, PubMed, EMBASE, Econlit and Web of Science and NHSEED. We also inspected references of all relevant studies; and searched trials registers (ClinicalTrials.gov). Search terms used included cost, return on investment, economic, arts, music, storytelling, dancing, writing and older adult as well as social return on investment (SROI). The last search was performed on 09/11/2022. As many economic evaluations of ACIs (especially SROIs) are commissioned by government bodies or charitable organisations, a search of the grey literature was undertaken.

### Handling searches

A PRISMA (Preferred Reporting Items for Systematic Reviews and Meta-Analyses) flow chart was used to document study selection, illustrating the numbers of records retrieved and selection flow through the screening rounds [56–58]; all excluded records (with rationale for exclusion) were documented.

### Selection of studies

Two screening rounds were conducted independently by two health economists experienced in undertaking reviews (GC, CO’N). The first round screened the title and abstract of articles based on the eligibility criteria; those selected at this stage entered a second round of full text screening with eligibility based on the inclusion and exclusion criteria. Any disagreements were discussed among the two reviewers, with access to a third reviewer available to resolve disagreements, though this proved unnecessary.

### Data extraction and management

Two reviewers extracted relevant information independently using an proforma developed specifically for the purposes of this study, which included all 35 items suggested by Wijnen et al. (2016) [48]. Information was extracted in relation to the following factors: (1) general information including study title, author, year, funding source, country, setting and study design; (2) recruitment details, sample size, demographic characteristics (age, gender) and baseline health data (diagnosis, comorbidities); (3) interventions, effectiveness and cost data; (4) type of economic evaluation, perspective, payer, beneficiary, time horizon, measure of benefit and scale of intervention; (5) quality assessment, strength of evidence, any other important information; (6) results; (7) analysis of uncertainty and (8) conclusions. The quality assessment/risk of bias checklists were included in the data extraction proforma, and picklists were used to enhance uniformity of responses. The data extraction form was piloted by two reviewers (GC and CON) on one paper and discussion used to ensure consistent application thereafter.

### Assessment of study quality

Two reviewers (GC & CON) independently assessed study quality, with recourse to a third reviewer for resolution of differences though this proved unnecessary. Quality assessment was based on the type of economic evaluation undertaken. Full and partial trial-based economic evaluations were assessed using the CHEC-extended checklist [59]. SROI analyses were assessed using a SROI-specific quality framework developed for the purpose of systematic review [60].

### Data analysis methods

Due to the small number of evaluations detected, possible sources of heterogeneity and a lack of consensus on appropriate methods for pooling cost-effectiveness estimates [61] a narrative synthesis analysis was undertaken.

## Results

**Fig. 1:**
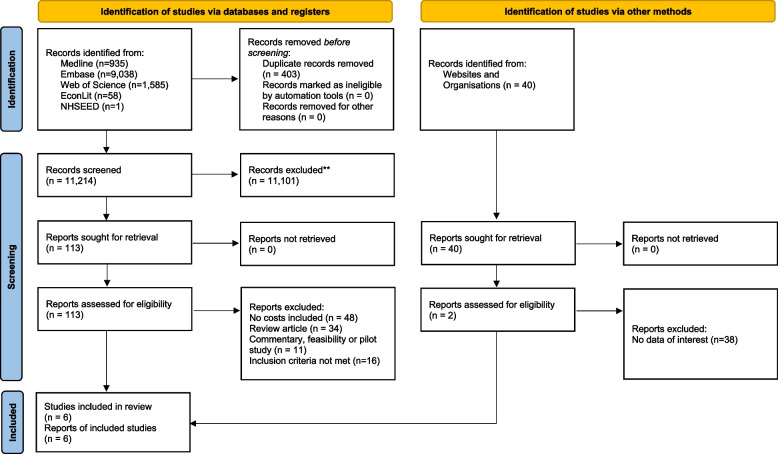
PRISMA 2020 flow diagram for new systematic reviews which include searches of databases, registers and other sources

Database searches returned 11,619 records; from this, 402 duplicates were removed leaving 11,214 reports. From these 113 reports were assessment against the inclusion and exclusion criteria resulting in 4 studies for inclusion in the review. Over 40 websites were searched for relevant content returning 2 further studies for inclusion. The PRISMA 2020 diagram is presented in Fig. 1. A high sensitivity search strategy was adopted to ensure all relevant studies were identified, resulting in a large number of studies being excluded at the first stage of screening.

A total of six studies were identified; key characteristics are presented in Table 1. Identified studies were published between 2011 and 2020. Two studies used a health technology assessment (HTA) framework alongside clinical trials [62, 63] to assess the cost-effectiveness of community singing interventions. Both evaluations scored highly on the CHEC-extended checklist (Table 2), with findings reported in line with the CHEERS (Consolidated Health Economic Estimation Reporting Standards) checklist 2022 [64].

**Table 1:** Details of economic studies included in the review

	**"Silver Song Club" programme**	**"Community of Voices" trial**	**The Imagine Arts programme**	**"Dementia and imagination" programme**	**"Craft Café"**	**"Creative Caring"**
**Title**	Coulton et al., 2015 [62]	Johnson et al., 2020 [63]	Bosco et al., 2019 [65]	Jones et al., 2020 [66]	Social Value Lab, 2011 [67]	MB Associates, 2013 [68]
**Programme**	Singing	Singing	Art programme	Art programme	Art and craft programme	Craft programme
**Intervention**	14-week, 90-min programme	44-week, 90-min choir sessions	12 months- attendance of 1 session specified	12 week, 2-h sessions	12-week programme	12-month programme in care home
**Population**	Older adults	Older adults	Older adults with or without dementia	Older adults with dementia and caregivers	Older adults	Older adults
**Setting**	Community	Community	Care home	Hospital, community, residential	Community	Care home
**Sample size**	258 participants	390 participants	267 participants	125 participants	Two housing associations	Five care homes
**Location**	UK	USA	UK	UK	UK	UK
**Economic evaluation**	CEA	Outcome evaluation (including healthcare utilisation)	SROI	SROI	SROI	SROI
**Underlying study design**	RCT	RCT	Qualitative study	Mixed methods longitudinal study	Pilot study	Pilot study
**Study perspective**	The payer	The payer	Participants	Range of stakeholders	Range of stakeholders	Range of stakeholders
**Comparator(s)**	Usual care	Wait-list control	No comparator	No comparator	No comparator	no comparator
**Time horizon**	6 months (extrapolated to 12 months)	6 months	12 months (extrapolated to 4 years)	6 months (extrapolated to 12 months)	12 months (extrapolated to 3 years)	12 months (extrapolated to 2 years)
**Discount rate**	n/a	n/a	3.5%	3.5%	3.5%	n/a
**Health and QoL related outcomes**	Health related Quality of life; mental health QoL, anxiety and depression	Psychosocial, cognitive and physical outcomes; health care utilisation and costs	Social isolation, quality in mental health and mobility, community inclusion and cognition	Wellbeing and mood; confidence; control; social isolation, physical activity	Physical exercise, loneliness, anxiety and depression, harmful behaviours e.g. smoking	Mentally active and independent; relationships
**Measurement of health and QoL related outcomes**	QALY (EQ-5D)	PHQ-8; TMT; The NIH Toolbox; healthcare cost questionnaire	Qualitative data from reflective diaries; and range of assumptions	Specific questions on DEMQOL-proxy questionnaire	Qualitative data and range of assumptions	Forecasts based on qualitative data and range of assumptions
**Source of costs/financial proxies**	National sources	National sources	Range of assumptions	HACT social value bank; range of Assumptions	Range of assumptions	Range of assumptions
**Uncertainty**	CEAC	Statistical analysis	sensitivity analysis (assumptions)	sensitivity analysis (assumptions)	sensitivity analysis (assumptions)	sensitivity analysis (assumptions)
**Presentation of results**	CEAC	statistical analysis	SROI ratios	SROI ratios	SROI ratios	SROI ratios
**Conclusions**	64% probability of being cost-effective at £30,000	No significant differences in primary outcomes (including healthcare utilisation)	SROI £1.20: £1	SROI £3.20-£6.62: £1	SROI £ 4.86-£9.57: £1	SROI £3: £1

**Table 2:** CHEC Quality Assessment Checklist

	**Title**	**Coulton et al., (2015) **[62]	**Johnston et al., (2018)**
		Silver Song Club	Community of Voices
**1**	Is the study population clearly described?	✓	✓
**2**	Are competing alternatives clearly described?	✓	✓
**3**	Is a well-defined research question posed in an answerable form?	✓	✓
**4**	Is the economic study design appropriate to the stated objective?	✓	✓
**5**	Is the chosen time horizon appropriate to include relevant costs and consequences?	✓	✓
**6**	Is the actual perspective chosen appropriate?	✓	✓
**7**	Are all important and relevant costs for each alternative identified?	✓	✓
**8**	Are all costs measured appropriately in physical units?	✓	✓
**9**	Are costs valued appropriately?	✓	✓
**10**	Are all important and relevant outcomes for each alternative identified?	✓	✓
**11**	Are all outcomes measured appropriately?	✓	✓
**12**	Are outcomes valued appropriately?	✓	✓
**13**	Is an incremental analysis of costs and outcomes of alternatives performed?	✓	x^a^
**14**	Are all future costs and outcomes discounted appropriately?	✓	✓
**15**	Are all important variables, whose values are uncertain, appropriately subjected to sensitivity analysis?	✓	✓
**16**	Do the conclusions follow from the data reported?	✓	✓
**17**	Does the study discuss the generalizability of the results to other settings and patient/client groups?	✓	✓
**18**	Does the article indicate that there is no potential conflict of interest of study researcher(s) and funder(s)?	✓	✓
**19**	Are ethical and distributional issues discussed appropriately?	✓	✓

^a^No difference between groups therefore no need to perform Incremental cost effectiveness analysis

Four further studies employed an SROI framework to assess art and/or craft interventions: two studies were published in the peer-reviewed literature [65, 66] and a further two in the grey literature [67, 68]. All four adhered closely to the suggested steps for performing an SROI and consequently secured high scores (Table 3). No quality differential was discerned between those studies published in the academic literature when compared with those from the grey literature.

**Table 3:**
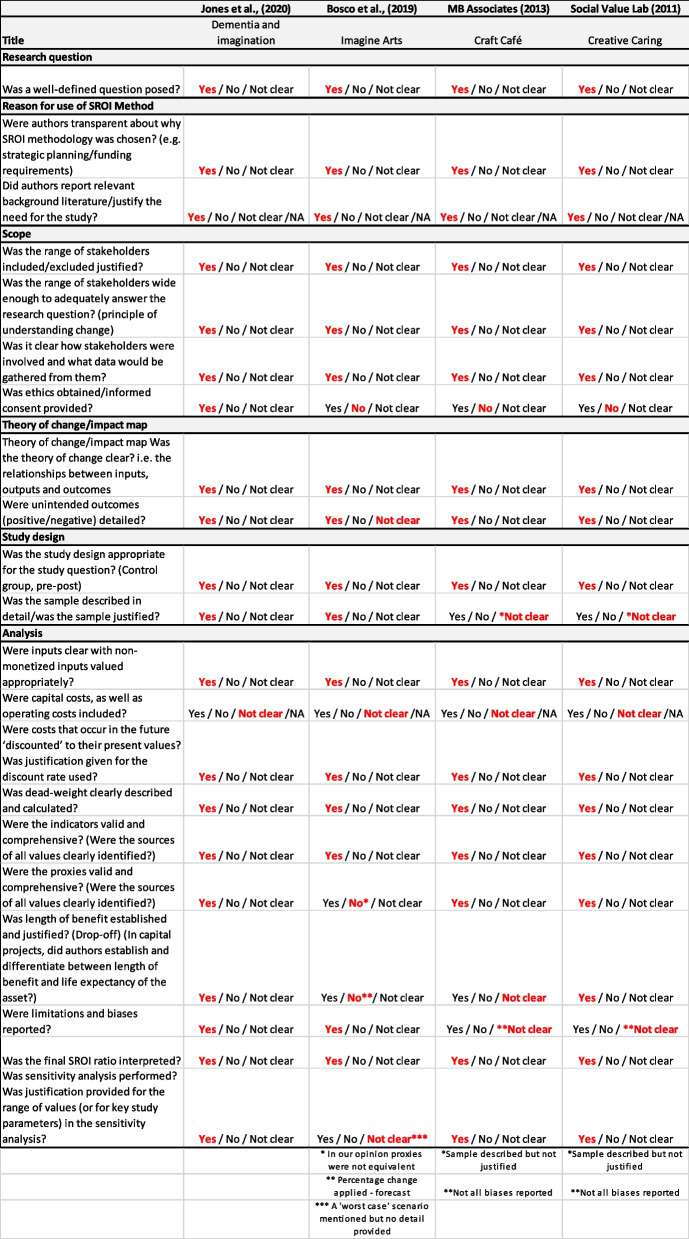
SROI checklist

Five of the studies were undertaken in the UK [63, 66–69] and one in the US [63]. Four of the studies were designed for older adults with no cognitive impairment [62, 63, 67, 68]; one was designed for participants with or without dementia [65], and another was specifically for older adults with dementia and their caregivers [66]. Three of the studies were delivered in a community setting [62, 63, 67], two in care homes [65, 68] and one across a range of settings (hospital, community and residential) [66]. The length and duration of the ACIs varied; some lasted 1–2 h (with multiple classes available to participants) [65], whereas others were structured programmes with sessions lasting 90 min over a 14-week period [62]. The number of participants included in studies varied; the largest study contained data from 390 participants [63], whereas other studies measured engagement using numbers of care homes or housing associations included [67, 68].

Costs were captured from a narrower perspective (i.e., the payer—health service) for those economic evaluations which followed a health technology assessment (HTA) framework [62, 63]. Costs associated with providing the programme and health and social care utilisation costs were captured using cost diaries. Valuation of resource usage was in line with the reference case specified for each jurisdiction.

Social value analyses included in the review [65–68] captured a broader picture of cost; programme provision costs included were similar in nature to those identified using an HTA framework, however, the benefits captured went beyond the individual to capture costs to a wide range of stakeholders such as family members, activity co-ordinations and care home personnel. Costs were apportioned using financial proxies from a range of sources including HACT Social Value Bank [69] and market-based valuation methods.

The range of outcomes captured and valued across HTAs and SROIs was extensive: including, but not limited to, wellbeing, quality of life, physical health, cognitive functioning, communication, control over daily life choices, engagement and empowerment, social isolation, mobility, community inclusion, depressive symptoms, sadness, anxiety, loneliness, positive affect and interest in daily life. In the programmes assessed using an HTA framework, outcomes were captured using standardised and validated instruments, for both control and intervention groups across multiple time points. Statistical methods were used to assess changes in outcomes over time. Programmes assessed using SROI relied primarily on qualitative methods (such as reflective diaries and in-depth interviews) combined with routinely collected administrative data. 

The evidence from the singing interventions was encouraging but not conclusive. The ‘Silver Song Club’ programme [62] reported a 64% probability of being cost-effective at a willingness-to-pay threshold of £30,000. This study was also included in the Public Health England (PHE) decision tool to support local commissioners in designing and implementing services to support older people’s healthy ageing, reporting a positive societal return on investment [70]. Evidence from the ‘Community of Voices’ trial [63] suggested that although intervention group members experienced statistically significant improvements in loneliness and interest in life compared to control participants, no significant group differences were observed for cognitive or physical outcomes or for healthcare costs.

A positive return on investment was reported by all social value analyses undertaken. The ‘Imagine Arts’ programme, reported a positive SROI of £1.20 for every £1 of expenditure [65]. A higher yield of between £3.20-£6.62 for each £1 invested was reported in the ‘Dementia and Imagination’ programme [66]. The ‘Craft Café’ programme, reported an SROI of £8.27 per £1 invested [68], and the ‘Creative Caring’ programme *predicted* a SROI of between £3 to £4 for every £1 spent [67]. The time period over which return on investment was calculated differed for each evaluation from less than one year to 4 years.

## Discussion

The primary finding from our review concerns the paucity of evidence relating to the value, cost and/or cost-effectiveness of ACIs aimed at improving health and wellbeing in this population. Despite few restrictions being applied to our search, only six studies were found which met our inclusion criteria. This is not indicative of research into ACIs in this population, as evidenced by the identification of ninety-three studies where arts and creativity interventions were found to support better health and wellbeing outcomes in another recent review [5]. An alternative explanation is that funders do not see the added value of undertaking such evaluations in this area. That is, for funders, the cost of evaluating an ACIs is likely to be deemed unjustified given the relatively small welfare loss a misallocation of resources to them might produce. While at first glance this may seem reasonable, it disadvantages ACIs in competing with other interventions for funding and arguably exposes an implicit prejudice in the treatment of interventions from which it may be difficult to extract profit in general. That is, the paucity of evidence, may reflect inherent biases within our political economy that favour the generation of marketable solutions to health issues from which value can be appropriated as profit. Pharmaceuticals are an obvious example of such solutions, where the literature is replete with examples of evaluations sponsored by pharmaceutical companies or where public funds are used to test the claims made by pharmaceutical companies in respect of the value of their products. If the potential of ACIs to improve health and well-being is to be robustly established, ACIs must effectively compete for funding with other interventions including those from pharma. This requires a larger, more robust evidence base than is currently available and investment in the creation of such an evidence base. As there is currently no ‘for-profit’ industry to generate such an evidence base, public funding of evaluations will be central to its creation.

Our second finding concerns the values reported in the meagre evidence we did find. In five of the six studies we identified, evidence indicated that ACIs targeted at older people offered value for money [62, 65–68]. One study provided mixed evidence [63], however, in this study a ‘payer’ perspective was adopted when applying an HTA framework which, by virtue of the perspective adopted, excluded a range of benefits attributable to ACIs and public health interventions more generally. Among the four studies that adopted a SROI approach, estimated returns per £1 invested ranged from £1.20 to £8.27. Given the evident heterogeneity among studies in terms of context and methods, care is warranted in comparing estimates with each other or with other SROIs. Care is also required in accepting at face value the estimates reported given methodological issues that pertain to the current state of the art with respect to SROI. With these caveats in mind noted, the values reported for ACIs using the SROI approach are comparable with those from other SROI studies in other contexts including those as diverse as a first aid intervention [71], investment in urban greenways [72] and the provision of refuge services to those experiencing domestic violence [73] (a return on investment of £3.50-£4, £2.88-£5.81 and £4.94 respectively). Similarly, with respect to the study that adopted a cost-effectiveness approach, Coulton and colleagues (2015) reported a 64% probability of the intervention being cost-effective at a threshold of £30,000 [62]. Again, it is difficult to compare studies directly, but this is similar to that reported for interventions as diverse as a falls prevention initiative [74] and the treatment of depression using a collaborative approach [75] both in the UK. That the evidence base is meagre notwithstanding, there is, in other words, a prima facie case that ACIs are capable of offering value for money when targeted at older persons.

Our third finding relates to the state of the art with respect to SROIs in this area. Over the past 40 years, considerable time, effort and resources have been expended in the development of cost-effectiveness techniques in health and social care. While considerable heterogeneity can exist around their conduct, national guidance exists in many jurisdictions on the conduct of cost-effectiveness analyses (CEA) – such as the NICE reference case in the UK [76]– as well as in the reporting of these as set out in the CHEERS 2022 guidance [64]. This has helped raise the quality of published evaluations and the consistency with which they are reported. Despite the existence of a step-by-step guidance document on how to perform SROIs [77] which outlines how displacement effects, double counting, effect attribution and drop-off should be addressed, a significant body of work still remains to ensure that the methodology addresses a range of known biases in a robust manner. Where there is no comparator to the intervention being evaluated (as was the case in the SROIs reported here) it may be difficult to convince funders that the implicit incremental costs and benefits reported are indeed incremental and attributable to the intervention. Equally, where a comparator is present, greater consensus and standardisation is required regarding the identification, generation and application of, for example, financial proxies. Currently, SROI ratios combine value across a wide range of stakeholders, which is understandable if the objective is to capture all aspects of social benefit generated. This ratio, however, may not reflect the priorities and statutory responsibilities of healthcare funders. Whist all of the aforementioned issues can be addressed, investment is required to develop the SROI methodology further to more closely meet the needs of commissioning bodies.

Notwithstanding these challenges, social value analyses play a pivotal role within the procurement processes employed by government, local authorities and other non-departmental public bodies and should not be dismissed simply because the ‘burden of proof’ falls short of that required to secure remuneration within the health sector. As most SROIs are published in the grey literature, this means they often avoid peer scrutiny prior to publication and the potential quality assurance this can offer. It is noteworthy however that two of the SROIs included in this review [65, 66] were published in the academic literature, suggesting that the academic community are engaging with this method which is to be applauded.

Moving forward, it is unlikely we will be able to meet all of the health and wellbeing needs of our ageing population solely in a primary or secondary care setting. New models of care are required, as are new models of funding to support interventions which can be delivered in non-healthcare settings. New hybrid models of evaluation will be required to provide robust economic evidence to assist in the allocation of scarce resources across health and non-healthcare settings; such evaluative frameworks must have robust theoretical underpinnings and be capable of delivering evidence from a non-clinical setting in a timely and cost-effective manner.

In the absence of a definitive evaluation framework for ACIs being currently available, we have a number of recommendations. First, and most importantly, all impact assessments should have a control group or credible counterfactual. This is currently not required when performing an SROI making it difficult to determine if all of the benefits ascribed to an intervention are in fact attributable. This recommendation is in line with the conclusion of a report by the London School of Economics [78] for the National Audit Office (NAO) which concluded that ‘any impact evaluation (and subsequent value for money calculation) *requires* construction of a counterfactual’. Second, a detailed technical appendix should accompany all impact assessments to allow independent review by a subject specialist. While this would assist peer review, it would allow providing greater transparency where peer review was not undertaken prior to publication. Furthermore, it would enable recalculation of SROI ratios to exclude ‘value’ attributable to stakeholders which are not relevant to a particular funder. Third, equity considerations should be addressed explicitly in all evaluations (this is currently not required in HTAs). Fourth, both costs and outcomes should be captured from a ‘broad’ perspective (adopting a ‘narrow’ healthcare perspective may underestimate the full economic impact), with non-healthcare sector costs being detailed as part of the analysis. Finally, data should be collected post-implementation to ensure that resources continue to be allocated efficiently.

As with any review, there are limitations which should be noted. A search of the grey literature was included as evaluations of applied public health interventions are not always reported in the academic literature. Systematically identifying grey literature and grey data can be problematic [79–83] as it is not collected, organised or stored in a consistent manner. Hence it is possible that we have not identified all relevant studies. Furthermore, as applied public health interventions can be performed in a non-healthcare setting we included SROIs in our review of economic evaluations. Current guidance on the systematic review of economic evaluations has been developed primarily for review of HTA as opposed to public health interventions and hence SROIs would be excluded, or if included would score poorly due to the inherent biases arising from no comparator or counterfactual being included.

## Conclusions

This systematic review found that participation in group-based arts and creativity programmes was generally cost-effective and/or produced a positive return on investment whilst having a positive impact on older people’s physical, psychological, and social health and wellbeing outcomes. Unfortunately, the small number of studies identified, coupled with differences in methods used to assess economic impact hinders our ability to conclusively determine which types of art and creativity-based activities are more cost-effective or represent best value for money.

As well as the need for a greater focus on prevention of poor health as we age, new hybrid models of healthcare delivery are necessary to meet the needs of our ageing population. These models will integrate traditional medical care with other services such as home health aides (some of which may include artificial intelligence), telemedicine and social support networks. Alongside these, ACIs have the potential to provide a low cost, scalable, easily implementable and cost-effective solution to reduce the burden of illness in this age group and support healthy ageing.

Evidence on the cost-effectiveness of a range of ACIs is of utmost importance for policy and decision makers as it can both inform the development of policies that support the provision of ACIs in the context of ageing, but also identify the most cost-effective approaches for delivering such interventions. The development of hybrid models of evaluation, capable of capturing cost-effectiveness and social value, is becoming increasingly necessary as healthcare delivery for this age group moves beyond the realms of primary and secondary care and into the community. The development and refinement of such models will ensure a more comprehensive assessment of the impact of a diverse range of interventions providing a more nuanced understanding of the impact of an intervention. This will help inform decision making and ensure interventions are implemented in a cost-effective and socially beneficial manner.

### Supplementary Information

**Additional file 1: Table S1.** Search strategy for electronic databases and grey literature.

## Data Availability

All data generated or analysed during this study are included in the published article and its supplementary information files.
