# Supplementary material for: Arts and creativity interventions for improving health and wellbeing in older adults: a systematic literature review of economic evaluation studies
Source: BMC Public Health. 2023 Dec 13;23:2496. doi: 10.1186/s12889-023-17369-x (PMC10717503; doi:10.1186/s12889-023-17369-x)
Supplement: Supplementary file 1 — Additional file 1: Table S1. Search strategy for electronic databases and grey literature. [file 12889_2023_17369_MOESM1_ESM.docx]

**Table S1: Search strategy for electronic databases and grey literature**

**Search string for Medline**

| #1 | (cost* or econ* or SROI or "social return on investment" or "social value" or SCBA or "social cost benefit analysis").ab,ti. |
| --- | --- |
| #2 | Economics/ |
| #3 | 1 or 2 |
| #4 | (Art or Arts or artist* or "art work" or music* or creativ* or sing* or choir or choral or painting or writing or "story telling" or Storytelling or poetry or drama* or theatr* or sculptur* or "film making" or "photography" or "performing art" or "spoken word" or "Arts on referral" or "Art on referral" or "social prescribing" or craft or "arts on prescription" or danc*).ab,ti. |
| #5 Art | Art Therapy/ or Art/ |
| #6 | Dancing/ |
| #7 | Writing/ |
| #8 | Museums/ |
| #9 | Photography/ |
| #10 | Poetry as Topic |
| #11 | Narration/ |
| #12 | 4 or 5 or 6 or 7 or 8 or 9 or 10 or 11 |
| #13 | (Memory or "well-being" or "well being" or wellbeing or "quality of life" or "social cohesion" or loneliness or anxiety or "self esteem" or "social inclusion" or "social engagement" or "social isolation" or "cognitive function" or "self-esteem" or "community health" or "psychological health" or "social health" or "social cohesion" or happy or happiness).ab,ti. |
| #14 | Quality of Life/ |
| #15 | Happines/ |
| #16 | Social Networking/ |
| #17 | Personal Autonomy/ |
| #18 | Social Inclusion/ |
| #19 | Social Participation/ |
| #20 | ("older adult*" or aging* or ageing* or cognition or frail* or older or senior or pensioner or elder* or "later life" or "third age" or "old* age" or "senior citizen" or geriatr* or dementia or alzheimer*).ab,ti. |
| #21 | A Community Choir Intervention to Promote Well-Being Among Diverse Older Adults: Results From the Community of Voices Trial.af. |
| #22 | Effectiveness and cost-effectiveness of community singing on mental health-related quality of life of older people: randomised controlled trial.ti. |
| #23 | Dementia and Imagination: A Social Return on Investment Analysis Framework for Art Activities for People Living With Dementia.ti. |
| #24 | The social value of the arts for care home residents in England.ti. |
| #25 | 21 or 22 or 23 or 24 |
| #26 | 13 or 14 or 15 or 16 or 17 or 18 or 19 |
| #27 | 3 and 12 and 20 and 26 |
| #28 | limit 27 to humans |
| #29 | protocol.m_titl. |
| #30 | 28 not 29 |
| #31 | 25 and 30 |
|  |  |

**Search string for EMBASE**

| #1 | cost/ or cost*.mp. |
| --- | --- |
| #2 | economics/ or econ*.mp. |
| #3 | social return on investment.mp. |
| #4 | sroi.mp. |
| #5 | social value/ |
| #6 | social cost benefit analysis.mp. [mp=title, abstract, heading word, drug trade name, original title, device manufacturer, drug manufacturer, device trade name, keyword heading word, floating subheading word, candidate term word] |
| #7 | scba.mp. [mp=title, abstract, heading word, drug trade name, original title, device manufacturer, drug manufacturer, device trade name, keyword heading word, floating subheading word, candidate term word] |
| #8 | art/ |
| #9 | arts.mp. |
| #10 | artist*.mp. |
| #11 | (art work or artwork).mp. [mp=title, abstract, heading word, drug trade name, original title, device manufacturer, drug manufacturer, device trade name, keyword heading word, floating subheading word, candidate term word] |
| #12 | music/ |
| #13 | creativity/ |
| #14 | singing/ |
| #15 | choir (singing)/ |
| #16 | choral.mp. |
| #17 | painting/ |
| #18 | writing/ |
| #19 | storytelling/ |
| #20 | literature/ |
| #21 | (poem* or poet*).mp. [mp=title, abstract, heading word, drug trade name, original title, device manufacturer, drug manufacturer, device trade name, keyword heading word, floating subheading word, candidate term word] |
| #22 | drama*.mp. [mp=title, abstract, heading word, drug trade name, original title, device manufacturer, drug manufacturer, device trade name, keyword heading word, floating subheading word, candidate term word] |
| #23 | (theatr* or theater*).mp. [mp=title, abstract, heading word, drug trade name, original title, device manufacturer, drug manufacturer, device trade name, keyword heading word, floating subheading word, candidate term word] |
| #24 | sculptur*.mp. [mp=title, abstract, heading word, drug trade name, original title, device manufacturer, drug manufacturer, device trade name, keyword heading word, floating subheading word, candidate term word] |
| #25 | movie/ |
| #26 | film?mak*.mp. [mp=title, abstract, heading word, drug trade name, original title, device manufacturer, drug manufacturer, device trade name, keyword heading word, floating subheading word, candidate term word] |
| #27 | photography/ |
| #28 | performing arts/ or performing artist/ |
| #29 | spoken word.mp. |
| #30 | art* on referral.mp. |
| #31 | social prescribing.mp. |
| #32 | (craft or crafts).mp. [mp=title, abstract, heading word, drug trade name, original title, device manufacturer, drug manufacturer, device trade name, keyword heading word, floating subheading word, candidate term word] |
| #33 | art* on prescription.mp. |
| #34 | dancing/ |
| #35 | art therapy/ |
| #36 | information center/ |
| #37 | museum*.mp. |
| #38 | narrative/ |
| #39 | memory/ |
| #40 | wellbeing/ |
| #41 | quality of life/ |
| #42 | social cohesion/ |
| #43 | loneliness/ |
| #44 | anxiety/ |
| #45 | self esteem/ |
| #46 | social inclusion/ |
| #47 | social engagement.mp. |
| #48 | social isolation/ |
| #49 | cognitive function.mp. or cognition/ |
| #50 | community health.mp. |
| #51 | psychological well-being/ or psychological health.mp. |
| #52 | social health.mp. |
| #53 | social cohesion/ |
| #54 | happiness/ |
| #55 | happy.mp. |
| #56 | social networking.mp. or social network/ |
| #57 | personal autonomy/ |
| #58 | social participation/ |
| #59 | aged/ |
| #60 | older adult*.mp. |
| #61 | aging/ |
| #62 | ageing.mp. |
| #63 | cognition/ |
| #64 | frailty/ |
| #65 | older.mp. |
| #66 | senior.mp. |
| #67 | pensioner/ |
| #68 | elderly.mp. |
| #69 | later life.mp. [mp=title, abstract, heading word, drug trade name, original title, device manufacturer, drug manufacturer, device trade name, keyword heading word, floating subheading word, candidate term word] |
| #70 | third age.mp. [mp=title, abstract, heading word, drug trade name, original title, device manufacturer, drug manufacturer, device trade name, keyword heading word, floating subheading word, candidate term word] |
| #71 | old age.mp. or senescence/ |
| #72 | geriatrics/ |
| #73 | dementia/ |
| #74 | Alzheimer disease/ |
| #75 | or/1-7 |
| #76 | or/8-38 |
| #77 | or/39-58 |
| #78 | or/59-74 |
| #79 | 75 and 76 and 77 and 78 |
| #80 | limit 79 to human |
| #81 | protocol.ti. |
| #82 | 80 not 81 |

**Search string for Web of Science**

| #1 | (TI=(cost* or econ* or SROI or "social return on investment" or "social value" or SCBA or "social cost benefit analysis") OR AB=(cost* or econ* or SROI or "social return on investment" or "social value" or SCBA or "social cost benefit analysis")) AND (TI=(Art or Arts or artist* or "art work" or music* or creativ* or sing* or choir or choral or painting or writing or "story telling" or Storytelling or poetry or drama* or theatr* or sculptur* or "film making" or "photography" or "performing art" or "spoken word" or "Arts on referral" or "Art on referral" or "social prescribing" or craft or "arts on prescription" or danc*) OR AB=(Art or Arts or artist* or "art work" or music* or creativ* or sing* or choir or choral or painting or writing or "story telling" or Storytelling or poetry or drama* or theatr* or sculptur* or "film making" or "photography" or "performing art" or "spoken word" or "Arts on referral" or "Art on referral" or "social prescribing" or craft or "arts on prescription" or danc*))  AND (TI=(Memory or "well-being" or "well being" or wellbeing or "quality of life" or "social cohesion" or loneliness or anxiety or "self esteem" or "social inclusion" or "social engagement" or "social isolation" or "cognitive function" or "self-esteem" or "community health" or "psychological health" or "social health" or "social cohesion" or happy or happiness) OR AB=(Memory or "well-being" or "well being" or wellbeing or "quality of life" or "social cohesion" or loneliness or anxiety or "self esteem" or "social inclusion" or "social engagement" or "social isolation" or "cognitive function" or "self-esteem" or "community health" or "psychological health" or "social health" or "social cohesion" or happy or happiness)) AND (TI=("older adult*" or aging* or ageing* or cognition or frail* or older or senior or pensioner or elder* or "later life" or "third age" or "old* age" or "senior citizen" or geriatr* or dementia or alzheimer*) OR AB=("older adult*" or aging* or ageing* or cognition or frail* or older or senior or pensioner or elder* or "later life" or "third age" or "old* age" or "senior citizen" or geriatr* or dementia or alzheimer*)) |
| --- | --- |
| #2 | TI=protocol |
| #3 | #1 NOT #2 |
| #4 | (TI="A Community Choir Intervention to Promote Well-Being Among Diverse Older Adults: Results From the Community of Voices Trial") OR (TI="Effectiveness and cost-effectiveness of community singing on mental health-related quality of life of older people: randomised controlled trial") OR (TI="Dementia and Imagination: A Social Return on Investment Analysis Framework for Art Activities for People Living With Dementia") OR (TI="The social value of the arts for care home residents in England") |
| #5 | #3 AND #4 |

**Search string for EconLit**

| #1 | AB ( (cost* or econ* or SROI or "social return on investment" or "social value" or SCBA or "social cost benefit analysis") ) OR TI ( (cost* or econ* or SROI or "social return on investment" or "social value" or SCBA or "social cost benefit analysis") ) |
| --- | --- |
| #2 | AB ( (Art or Arts or artist* or "art work" or music* or creativ* or sing* or choir or choral or painting or writing or "story telling" or Storytelling or poetry or drama* or theatr* or sculptur* or "film making" or "photography" or "performing art" or "spoken word" or "Arts on referral" or "Art on referral" or "social prescribing" or craft or "arts on prescription" or danc*) ) OR TI ( (Art or Arts or artist* or "art work" or music* or creativ* or sing* or choir or choral or painting or writing or "story telling" or Storytelling or poetry or drama* or theatr* or sculptur* or "film making" or "photography" or "performing art" or "spoken word" or "Arts on referral" or "Art on referral" or "social prescribing" or craft or "arts on prescription" or danc*) ) |
| #3 | AB ( (Memory or "well-being" or "well being" or wellbeing or "quality of life" or "social cohesion" or loneliness or anxiety or "self esteem" or "social inclusion" or "social engagement" or "social isolation" or "cognitive function" or "self-esteem" or "community health" or "psychological health" or "social health" or "social cohesion" or happy or happiness) ) OR TI ( (Memory or "well-being" or "well being" or wellbeing or "quality of life" or "social cohesion" or loneliness or anxiety or "self esteem" or "social inclusion" or "social engagement" or "social isolation" or "cognitive function" or "self-esteem" or "community health" or "psychological health" or "social health" or "social cohesion" or happy or happiness) ) |
| #4 | AB ( ("older adult*" or aging* or ageing* or cognition or frail* or older or senior or pensioner or elder* or "later life" or "third age" or "old* age" or "senior citizen" or geriatr* or dementia or alzheimer*) ) OR TI ( ("older adult*" or aging* or ageing* or cognition or frail* or older or senior or pensioner or elder* or "later life" or "third age" or "old* age" or "senior citizen" or geriatr* or dementia or alzheimer*) ) |
| #5 | S1 AND S2 AND S3 AND S4 |
| #6 | TI protocol |
| #7 | S5 NOT S6 |
| #8 | TX "A Community Choir Intervention to Promote Well-Being Among Diverse Older Adults: Results From the Community of Voices Trial" OR TX ( "Effectiveness and cost-effectiveness of community singing on mental health-related quality of life of older people: randomised controlled trial" ) OR TX "The social value of the arts for care home residents in England" OR TX "A Social Return on Investment Analysis Framework for Art Activities for People Living With Dementia" |
|  |  |

**Search string for NHSEED**

| #1 | (Art or Arts or artist* or "art work" or music* or creativ* or sing* or choir or choral or painting or writing or "story telling" or Storytelling or poetry or drama* or theatr* or sculptur* or "film making" or "photography" or "performing art" or "spoken word" or "Arts on referral" or "Art on referral" or "social prescribing" or craft or "arts on prescription" or danc*):TI IN NHSEED |
| --- | --- |
| #2 | (cost* or econ* or SROI or "social return on investment" or "social value" or SCBA or "social cost benefit analysis"):TI IN NHSEED |
| #3 | (Memory or "well-being" or "well being" or wellbeing or "quality of life" or "social cohesion" or loneliness or anxiety or "self esteem" or "social inclusion" or "social engagement" or "social isolation" or "cognitive function" or "self-esteem" or "community health" or "psychological health" or "social health" or "social cohesion" or happy or happiness):TI IN NHSEED |
| #4 | ("older adult*" or aging* or ageing* or cognition or frail* or older or senior or pensioner or elder* or "later life" or "third age" or "old* age" or "senior citizen" or geriatr* or dementia or alzheimer*):TI IN NHSEED |
| #5 | MeSH DESCRIPTOR Economics EXPLODE ALL TREES |
| #6 | MeSH DESCRIPTOR Art EXPLODE ALL TREES |
| #7 | MeSH DESCRIPTOR Art Therapy EXPLODE ALL TREES |
| #8 | MeSH DESCRIPTOR Dancing EXPLODE ALL TREES |
| #9 | MeSH DESCRIPTOR Drawing EXPLODE ALL TREES |
| #10 | MeSH DESCRIPTOR Drawing EXPLODE ALL TREES |
| #11 | MeSH DESCRIPTOR Writing EXPLODE ALL TREES |
| #12 | MeSH DESCRIPTOR Museums EXPLODE ALL TREES |
| #13 | MeSH DESCRIPTOR Photography EXPLODE ALL TREES |
| #14 | MeSH DESCRIPTOR Poetry EXPLODE ALL TREES |
| #15 | MeSH DESCRIPTOR Quality of Life EXPLODE ALL TREES |
| #16 | MeSH DESCRIPTOR Happiness EXPLODE ALL TREES |
| #17 | MeSH DESCRIPTOR Social Networking EXPLODE ALL TREES |
| #18 | MeSH DESCRIPTOR Personal Autonomy EXPLODE ALL TREES |
| #19 | MeSH DESCRIPTOR Social Inclusion EXPLODE ALL TREES |
| #20 | MeSH DESCRIPTOR Social Participation EXPLODE ALL TREES |
| #21 | #2 OR #5 |
| #22 | #1 OR #6 OR #7 OR #8 OR #9 OR #10 OR #11 OR #12 OR #13 OR #14 |
| #23 | #3 OR #15 OR #16 OR #17 OR #18 OR #19 OR #20 |
| #24 | #4 AND #21 AND #22 AND #23 |
| #25 | (protocol):TI IN NHSEED |
| #26 | #24 NOT #25 |
| #27 | ("Effectiveness and cost-effectiveness of community singing on mental health-related quality of life of older people"):TI IN NHSEED |
| #28 | ("A Community Choir Intervention to Promote Well-Being Among Diverse Older Adults"):TI IN NHSEED |
| #29 | ("The social value of the arts for care home residents in England"):TI IN NHSEED |
| #30 | ("A Social Return on Investment Analysis Framework for Art Activities for People Living With Dementia"):TI IN NHSEED |
|  |  |

**Search for Grey Literature**

| \| [BASE](http://www.base-search.net/) \| \| --- \| \|  \| \| [British Library](http://explore.bl.uk/) \|  \| \| [CADTH](https://www.cadth.ca/) \|  \| \| [CASH](http://cash.libraryservices.nhs.uk/) \|  \| \| [COPAC](http://copac.jisc.ac.uk/) \|  \| \|  \|  \| \| [EThOS](http://ethos.bl.uk/Home.do;jsessionid=83730F6651FCDA26688497471FDA2EF3) \|  \| \| [King’s Fund](https://www.kingsfund.org.uk/) \|  \| \|  \|  \| \| [MedNar](http://mednar.com/mednar/desktop/en/search.html) \|  \| \| [NDLTD](http://search.ndltd.org/) \|  \| \|  \|  \| \| [Nuffield Trust](http://www.nuffieldtrust.org.uk/?gclid=CjwKEAiA-rfDBRDeyOybg8jd2U4SJAAoE5Xqvk5g2wDYf5ylRdvGcqkUcybryauoxffe_8nLhs7CpxoC9dzw_wcB) \|  \| \| [OAIster](http://oaister.worldcat.org/) \|  \| \| [OATD](https://oatd.org/) \|  \| \|  \|  \| \| [OPEN DOAR](http://www.opendoar.org/) \|  \| \| [OpenGrey](http://www.opengrey.eu/) \|  \| \| [PLOS](https://www.plos.org/) \|  \| \|  \|  \| \| [SCIE Online](https://www.scie.org.uk/) \|  \| \|  \|  \| \| [TRIPDatabase](http://www.tripdatabase.com/) \|  \| \| [UKHF](http://www.ukhealthforum.org.uk/) \|  \| | \| (Bielefeld Academy Search Engine) Bielefeld University Library tool.100 million documents – open access repository. \| \| --- \| \| Copyright Library. Main catalogue for millions of records for books, journals, newspapers, printed maps, scores, electronic resources, sound archive items etc. in the Library's collections. \| \| CADTH is an independent, not-for-profit organization responsible for providing health care decision-makers with objective evidence to help make informed decisions about the optimal use of health technologies, including: \| \| Current Awareness Service for Health \| \| Union catalogue of the major research libraries in the UK (including British Library and other copyright libraries. \| \| Seamless access to millions of open access research papers \| \| British Library - 400,000 doctoral theses. \| \| Independent views on health and social care. \| \| Reports, blogs and more \| \| Mednar is a free, medically-focused deep web search engine that uses Explorit Everywhere, an advanced search technology by Deep Web Technologies. As an alternative to Google, Mednar accelerates your research with a search of authoritative public and deep web resources, returning the most relevant results to one easily navigable page \| \| Theses and Dissertations \| \| 4,558,431 electronic theses and dissertations contained in the NDLTD archive. \| \| The Nuffield Trust is an independent health charity. Their aim is to improve the quality of health care in the UK by providing evidence-based research and policy analysis, and informing/generating debate. \| \| OAIster is a union catalogue of millions of records that represent open access resources, built through harvesting from collections worldwide. Includes more than 50 million records that represent digital resources from more than 2,000 contributors. \| \| Open Access Theses and Dissertations. \| \| Advanced research and scholarship. Theses and dissertations, free to find, free to use. \| \| *Open*DOAR is an authoritative directory of academic open access repositories. \| \| OpenGrey covers Science, Technology, Biomedical Science, Economics, Social Science and Humanities. \| \| Public Library of Science (PLOS) \| \| A non-profit Open Access publisher, innovator and advocacy organization with a mission to accelerate progress in science and medicine by leading a transformation in research communication. \| \| Social Care Institute for Excellence \| \| A leading improvement support agency and independent charity working with adult, family and children's care and support services across the UK. We also work closely with related services such as health care and housing. \| \| Trip is a clinical search engine, but as well as research evidence, it also provides a gateway to other content types, including images, videos, patient information leaflets, educational courses and news. \| \| UK Health Forum - Prevention Information & Evidence eLibrary \| |
| --- | --- | --- | --- | --- | --- | --- | --- | --- | --- | --- | --- | --- | --- | --- | --- | --- | --- | --- | --- | --- | --- | --- | --- | --- | --- | --- | --- | --- | --- | --- | --- | --- | --- | --- | --- | --- | --- | --- | --- | --- | --- | --- | --- | --- | --- | --- | --- | --- | --- | --- | --- | --- | --- | --- | --- | --- | --- | --- | --- | --- | --- | --- | --- | --- | --- | --- | --- | --- | --- | --- | --- | --- | --- |
| **Sources searched** | \|  \| \| --- \| \| Aesop \| \| All Parlimentary Group for Arts, Health and Wellbeing \| \| Big Lottery \| \| Charities Evaluation Service \| \| Creative and Credible \| \| Cultural Commissioning Programme \| \| National Institute for Health and Clinical Excellence (NICE) \| \| National Alliance for Arts, Health and Wellbeing \| \| NIHR Research Design Service \| \| New Economics Foundation \| \| Public Health Practice Evaluation Scheme \| \| Social Care Institute of Excellence (SCIE) \| \| What Works Centre for Wellbeing \| \| Willis Newson \| \| Arts Council \| \| The Welsh NHS Confederation - Advancing Arts, Health and Wellbeing \| \| Irish Arts Council \| \| SCHARRHud \| \| ASCOT \| \| ICECAP \| \| Campbell Collaboration Library \| \| Database of Abstracts of Reviews of Effects (DARE) \| \| Cochrane Database of  Systematic Reviews (CDSR) \| \| Public Health England (PHE) \| \| Personal Social Services Research Unit (PSSRU) \| \| Economics of Social and Healthcare Research Unit \| \| EPPI-Centre \| \| Age UK The older adults' NHS and social care return on investment tool \| \| Joseph Rowntree Foundation \| \| Association of Adult Social Services \| \| King's Fund \| \| Nuffield Trust \| \| Centre for Ageing and Development Research Ireland \| \| Institute for Research and Innovation in Social Services \| \| NIHR School of Social Care Research \| \| IPH \| \| CRD database \| |
|  |  |
